# Early Management Experience of Perforation after ERCP

**DOI:** 10.1155/2012/657418

**Published:** 2012-07-26

**Authors:** Guohua Li, Youxiang Chen, Xiaojiang Zhou, Nonghua Lv

**Affiliations:** Department of Gastroenteroloy, The First Affiliated Hospital of Nanchang University, Jiangxi Province, Nanchang 330006, China

## Abstract

*Background and Aim*. Perforation after endoscopic retrograde cholangiopancreatography (ERCP) is a rare complication, but it is associated with significant mortality. This study evaluated the early management experience of these perforations. *Patients and Methods*. Between November 2003 and December 2011, a total of 8504 ERCPs were performed at our regional endoscopy center. Sixteen perforations (0.45%) were identified and retrospectively reviewed. *Results*. Nine of these 16 patients with perforations were periampullary, 3 duodenal, 1 gastric fundus, and 3 patients had a perforation of an afferent limb of a Billroth II anastomosis. All patients with perforations were recognized during ERCP by X-ray and managed immediately. One patient with duodenal perforation and three patients with afferent limb perforation received surgery, others received medical conservative treatment which included suturing lesion, endoscopic nasobiliary drainage (ENBD), endoscopic retrograde pancreatic duct drainage (ERPD), gastrointestinal decompression, fasting, broad-spectrum antibiotics, and so on. All patients with perforation recovered successfully. *Conclusions*. We found that: (1) the diagnosis of perforation during ERCP may be easy, but you must pay attention to it. (2) Most retroperitoneal perforations can recover with only medical conservative treatment in early phase. (3) Most peritoneal perforations need surgery unless you can close the lesion up under endoscopy in early phase.

## 1. Introduction

Perforations related with endoscopic retrograde cholangiopancreatography (ERCP) are rare but serious complications. Its incidence has been reported by recent studies ranging from 0.3% to 2.1% [1]. Many patients with ERCP-related perforations recovered by surgery or by conservative therapy [2–6]. However, we do not know which patients require surgery, and when these patients should receive surgery. In this study we evaluate our experience for early management of ERCP-related perforations at our endoscopy centre.

## 2. Patients and Methods

A total of 8504 ERCPs were performed at our endoscopy centre (The Digestive Endoscopy Centre of Jiangxi Province) from November 2003 to December 2011. We looked retrospectively up all the cases in this period. A total of 16 perforations (0.19%) were identified. Patient demographics including age, sex, and comorbidities such as coronary heart disease (CHD), chronic obstructive pulmonary disease (COPD), chronic renal failure, and malignancy were noted. The indication for ERCP, clinical presentation, management, and length of stay in hospital were also recorded and analyzed.

## 3. Results

**Figure 1:**
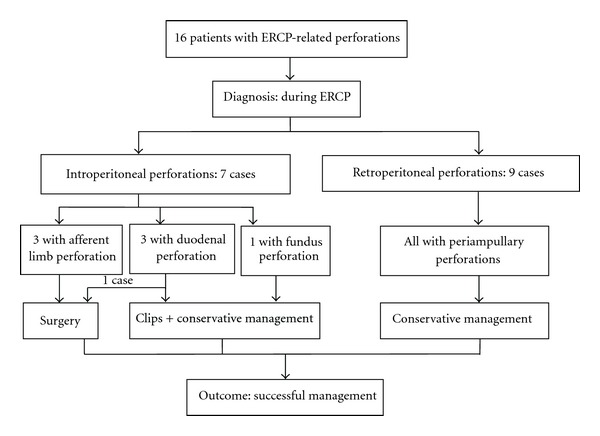
The management and outcome of 16 patients with ERCP-related perforation.

**Figure 2:**
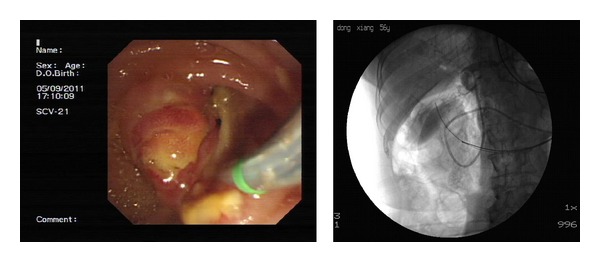
Pre-ampullary perforation by cutting. The kidney shadow was shown by X-ray.

**Figure 3:**
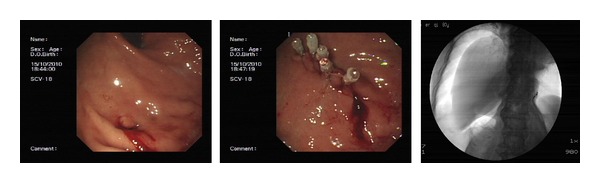
The fundus perforation was sutured by clips. The gas in peritoneal cavity was shown by X-ray.

**Figure 4:**
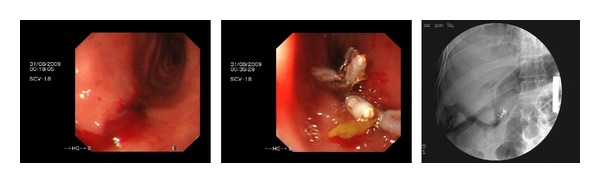
The duodenal lateral perforation was sutured by clips. The gas in peritoneal cavity was shown by X-ray.

Sixteen perforations were identified. The demographics, co-morbidities, ERCP indications, clinical presentation after ERCP perforation, management, and outcomes are presented in Table 1 and Figure 1. These included 1 fundus perforation (intraperitoneal perforation), 3 afferent limb perforations (intra-peritoneal perforation), 3 lateral wall of duodenal perforations (intra-peritoneal perforation), and 9 periampullaris perforations (retroperitoneal perforations). All perforations were diagnosed in the procedure of ERCP by X-ray fluoroscopy and/or endoscopy (Figures 2, 3, and 4). If we classifed the perforations as retroperitoneal perforations and peritoneal perforations, nine of them were retroperitoneal perforations, and the other seven were peritoneal perforations. Of the nine patients with retroperitoneal perforations, 5 resulted from papillotomy, 4 resulted from inserting balloon or basket into CBD after papillotomy during removing stone. These patients all suffered from CBD stones, and the stones were removed in the first ERCP attempt. After the initial ERCP, they were immediately treated with conservative management for 5 to 7 days. They all received ENBD, NG suction, fasting, intravenous nutrition, PPI, somatostatin (SS) and broad-spectrum antibiotics. Three patients received extraordinarily endoscopic retrograde pancreatic drainage (ERPD). Among them, one patient with Billroth II gastrectomy had preampullary perforation and incision bleeding. The incision bleeding may be related with taking NSAID for two years for treating arthrolithiasis. Another patient with preampullary perforation had mild acute ERCP-related pancreatitis. All of them recovered successfully by conservative management with an average length of stay in hospital of 12.6 days.

For the seven patients with peritoneal perforations, one patient had fundus perforation by duodenoscope when plugging it in. The perforation was diagnosed by duodenoscope and X-ray. The lesion was sutured immediately by five clips under gastroscope. The second ERCP attempt performed successfully 10 days later. Two patients with CBD stones had lateral wall of duodenum by duodenoscope when inserting duodenoscope. Both diagnosed immediately by X-ray and duodenoscope. The first attempt of ERCP in both patients stopped promptly. The lesions were sutured by clips under gastroscope. They received NG suction, PPI, SS, fasting, intravenous fluids, and broad-spectrum antibiotics for 7 days. One was shifted into surgery to remove CBD stones after perforation healing, and another was discharged due to being afraid of surgery and ERCP after perforation healing. The third patient's lateral duodenal wall perforation resulted from pushing duodenoscope during removing stone when basket captured the stone and passed extremity of common bile duct. The CBD stones of patients were removed in the initial ERCP, but the attempt of suturing the lesion by clips failed. The patient received immediately surgical operation with suturing lesion and abdominal cavity drainage. Three patients' afferent limb perforations resulted from plugging duodenoscope in, which were diagnosed by X-ray and endoscope during ERCP procedure. Among them, one patient suffered from cholangiocarcinoma. He received surgical operation with suturing lesion, abdominal cavity drainage, and CBD drainage (T tube drainage). Others received surgical operation with suturing lesion, removing CBD stones, and abdominal cavity drainage and CBD T-tube drainage. All patients, who received surgery, were treated with NG suction, PPI, SS, broad-spectrum antibiotic, fasting, and intravenous nutrition. All patients with peritoneal perforations have recovered successfully with an average length of stay in hospital of 18 days.

## 4. Discussion

ERCP has become important method for treating biliary-pancreatic diseases. However, the perforation related with ERCP is an infrequent, but severe complication. Its mortality could be as high as 37.5% [7, 8]. The reasons of perforation include patient-related factors (such as post Billroth II gastrectomy) and technique factors (such as inexperienced endoscopist, difficult cannulation, precut, and sphincterotomy) [8]. In our report, 7 lateral duodenal wall perforations resulted from duodenoscope injury, 5 peri-ampullary perforations resulted from papillotomy, and 4 peri-ampullary perforations resulted from inserting balloon or basket into CBD after papillotomy during removing stone. The main reason was technique factors. In order to reduce the perforation incidence, the high-risk patients should acquire experienced endoscopist to operate, and the operator should be careful and inexcitable.

The diagnosis of ERCP-related perforations had been reported during the ERCP procedure or several days after the ERCP procedure [7, 8]. The delayed diagnosis played an important role in high mortality of patients with ERCP-related perforations [8, 9]. So early diagnosis of the complication is very important. We think it may be easy if we pay attention to it. The presentation of retroperitoneal perforation showed skin emphysema, clear kidney shadow and unexplainable air shadow in fluoroscopy X-ray. The presentation of peritoneal perforation showed free gas shadow under diaphragm in fluoroscopy X-ray, visible gastrointestinal wall lesion under endoscope, and peritonitis. The CT scan could confirm further [8]. In our study, all perforations were diagnosed during ERCP procedure by X-ray fluoroscopy, and all peritoneal perforations were diagnosed by fluoroscopy and endoscope. We took a fluoroscopy for each patient before and after ERCP procedure in order to find if perforations happened in the procedure. We found that the fluoroscopy after the procedure could help to confirm further the imaging presentations of perforations, comparing with the fluoroscopy before and during the procedure. The operator usually concentrated on the procedure in the course of ERCP, and did not pay attention to those imaging presentations. So we think that the fluoroscopy after the procedure is very important.

The aim of early diagnosis was to acquire management in time. The immediate treatment for ERCP-related perforations affected its mortality [7, 8, 10–12]. There was no unified guideline to manipulate. So there has been controversy in the management of ERCP-related perforations. For retroperitoneal perforation, some authors [13] have advocated early operations for all endoscopic sphincterotomy (ES) perforation. There is increasing evidence that most retroperitoneal perforations could be managed without surgery [5, 6, 14, 15]. Doctors do not know which patients require surgery, and when these patients should receive surgery. In our study, all peri-ampullary perforations (retroperitoneal perforations) in early phase recovered successfully with conservative treatment, including ENBD, NG suction, fasting, intravenous nutrition, PPI, SS, and broad-spectrum antibiotics for 5 to 7 days. Our experience suggests that these peri-ampullary perforations could recover with this conservative treatment in the early phase. This could be due to two reasons: (1) the peri-ampullary perforation was small perforation and (2) the conservative management in early phase could alleviate the stimulation and secretion of gastric acid, bile, and pancreatic liquid. Some reported these perforations can recover also with conservative treatment such as ERBD [5]. In our opinion, ENBD had more advantage compared with ERBD. ENBD could connect with vacuum aspiration, and take bile away from the site of perforation. The NG suction, PPI, SS and fasting could also alleviate the stimulation by diminishing the secretion of gastric acid, bile, and pancreatic liquid. Of nine cases with peri-ampullary perforation, only 3 cases received ERPD. So ERPD was not imperative process for this perforation conservative management. ERPD might decrease the incidence of ERCP-related pancreatitis when the patient had multiple risk factors for post-ERCP pancreatitis [16]. All patients' stones in CBD were removed in the ERCP procedure. So, we can manage the perforation by conservative management after completing ERCP procedure when finding the peri-ampullary perforation.

Most authors [2, 3, 10] thought that the peritoneal perforations required surgery. There were increasing evidence that these perforations could suture by clips [6, 21]. In our series, seven patients had peritoneal perforations which were diagnosed during ERCP procedure. Of them, three patients' perforations were sealed by clips under endoscope. Other four patients shifted into surgery. All patients with peritoneal perforations were managed successfully too. So some gastric-duodenal wall perforations can suture by clips under endoscope. Our experience told us that these perforations required surgery unless you could suture immediately the lesion by endoscope, because these kinds of perforations were usually too big to recover by conservative management. Surgery should be selected if another ERCP may be difficult to treat the patient's disease, which must be treated immediately.

**Figure 5:**
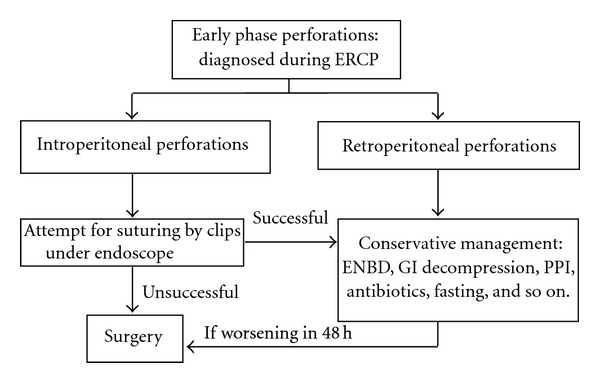
Early management algorithm of ERCP-related perforation.

Comparing our series of peri-ampullary perforations to that reported by others [8], we demonstrated a surprisingly low mortality. Our mortality rate was 0% versus 5%–37.5% in other's series [7, 8]. We believe that the superior results in our study were due to two factors: early diagnosis and effective management which reduced stimulation and exudation of gastric acid, bile, and pancreatic liquid in perforation site. We reviewed recent literature from the year 1999 onwards, which had more than 20 cases with ERCP-related perforation (Table 2). It was found that 87.9% retroperitoneal perforations could recover by conservative treatment (total mortality was 2.9%), and 80.8% peritoneal perforations received surgery (total mortality was 24.7%). This supports our opinion that most retroperitoneal perforations can recover only by conservative treatment, and most peritoneal perforations require surgery unless the perforation can be sutured under endoscope. At the same time, we found the death cause of 85.7% of patients with ERCP-related perforation was sepsis, and 81.8% patients who died had peritoneal perforation. Hence, it may be an important method to reduce mortality by sufficient drainage of exudation, and diminishing the exudation by reducing the secretion of gastric acid, bile, and pancreatic liquid. From our data and literature analysis, we suggest a protocol (Figure 5) as the early perforations management schedule. In our series, all patients with perforations were diagnosed immediately during ERCP procedure. The patients' peritoneal cavity and retroperitoneal space had little liquid exudation. If retroperitoneal space had large fluid exudation, the effective drainage is required by surgery or percutaneous puncturing drainage. We suggested the patient with perforation should shift to surgery if the patient was worsening within 48 h by conservative medicine, due to surgical operation having high mortality 48 h after perforation [21]. Although we had little experience for late perforations, the effective treatment should not only include the above management, but also include draining the fluid exudation by surgery or percutaneous puncturing drainage, preventing or treating infections by using broad-spectrum antibiotics, and early enteral nutrition (EEN) by placing nasojejunal catheter.

## Figures and Tables

**Table 1:** Patient demographics, ERCP indications, presentation and management of perforation, and outcome.

Age/sex	Comorbidities	ERCP indications	Type of ES	Clinical presentation	Type of perforation	Management	Length of stay (*d*)
56/M	Pancreatitis	CBD stones	—	Abdominal pain	Lateral duodenal perforation	Closure with six clips Surgery after perforation healing	23

72/F	COPD	CBD stones	—	Abdominal pain	Lateral duodenal perforation	Closure with five clips	14

88/M	HBP CAD	CBD stones	Standard	Peritonitis	Lateral duodenal perforation	Surgery (suture lesion and drainage abdominal cavity)	25

50/F	—	CBD stones	Standard	Emphysema	Retroperitoneal Perforation	ENBD Gastrointestinal decompression	12

58/M	—	CBD stones	Standard	Emphysema Abdominal pain (PEP)	Retroperitoneal Perforation	ENBD Gastrointestinal decompression	14

80/F	Pancreatitis HBP CAD	CBD stones	—	Symptomless for this perforation	Fundus perforation	Closure with five clips Next ERCP performed after 10 days	19

57/F	—	CBD stones	Pre-cut	Emphysema	Retroperitoneal Perforation	ENBD, ERPD, Gastrointestinal decompression	14

59/F	SAP HBP	CBD stones	Standard	Emphysema	Retroperitoneal Perforation	ENBD, ERPD Gastrointestinal decompression	22

67/M^∗^	Arthrolithiasis COPD, Billroth II gastrectomy	CBD stones	Standard	Abdominal pain Incision bleeding	Retroperitoneal Perforation	ENBD Gastrointestinal decompression	25

60/F	Pancreatitis HBP	CBD stones	Pre-cut	Symptomless	Retroperitoneal Perforation	ENBD Gastrointestinal decompression	7

60/M	COPD	Cholangio-carcinoma	—	Peritonitis	Afferent limb perforation	Surgery (suture lesion and drainage abdominal cavity and CBD)	18

53/F	—	CBD stones	—	Peritonitis	Afferent limb perforation	Surgery (suture lesion, T-tube drainage after removing CBD stones, and drainage of abdominal cavity)	14

56/M	Diabetes	CBD stones	—	Peritonitis	Afferent limb perforation	Surgery (suture lesion, T-tube drainage after removing CBD stones, and drainage of abdominal cavity)	13

63/F	—	CBD stones	Pre-cut	Emphysema	Retroperitoneal Perforation	ENBD, ERPD Gastrointestinal decompression	7

65/M	—	CBD stones	Standard	Emphysema	Retroperitoneal Perforation	ENBD Gastrointestinal decompression	7

58/F	—	CBD stones	Standard	Symptomless	Retroperitoneal Perforation	ENBD Gastrointestinal decompression	6

The conservative treatment included ENBD, NG suction, fasting, intravenous fluids, PPI, somatostatin (SS) and broad-spectrum antibiotics for 5 to 7 days. ^∗^The patient had to take NSAID for two years, and had complicated preampullary perforation and incision bleeding. The incision bleeding stopped by conservative treatment through adding antihemorrhagic 24 h after perforation.

**Table 2:** The perforations management and mortality in recent literature.

Author	No. of cases	Retroperitoneal perforation (surgery/died)	Peritoneal perforation (surgery/died)	Surgery treatment (%)	Mortality (%)	Died by sepsis
Ercan et al. [7]	24	6 (6/0)	18 (18/9)^∗^	24 (100)	9 (37.5)	6
Morgan et al. [11]	24	12 (0/0)	12 (10/1)	10 (41.6)	1 (7.1)	1
Fatima et al. [17]	75	41 (0/0)	34 (22/5)	22 (29.3)	5 (6.7)	5
Assalia et al. [18]	22	20 (2/1)	2 (2/0)	4 (18.2)	1 (4.5)	1
Wu et al. [19]	28	25 (5/2)	3 (3/2)	10 (35.7)	4 (14.3)	4
Thomas et al. [20]	40	36 (4/1)	4 (4/1)	8 (20)	2 (5)	2

Total	213	140 (17/4)	73 (59^#^/18^§^)	78 (36.7)	22 (10.3)	19

^∗^Having a esophagus perforation. ^§^The mortality of peritoneal perforation was more than that of retroperforation perforation (*P* = 0.000, chi square test). ^#^The rate of surgery treatment for peritoneal perforation was more than that for retroperforation perforation (*P* = 0.000, chi square test).
